# Association between Low Levels of Mannan-Binding Lectin and Markers of Autoimmune Thyroid Disease in Pregnancy

**DOI:** 10.1371/journal.pone.0081755

**Published:** 2013-12-10

**Authors:** Eliska Potlukova, Tomas Freiberger, Zdenka Limanova, Jan Jiskra, Zdenek Telicka, Jana Bartakova, Drahomira Springer, Hana Vitkova, Marten Trendelenburg

**Affiliations:** 1 Third Department of Medicine, General University Hospital and First Faculty of Medicine, Charles University in Prague, Prague, Czech Republic; 2 Centre for Cardiovascular Surgery and Transplantation, Molecular Genetics Laboratory, Brno, Czech Republic, and Ceitec and Faculty of Medicine, Masaryk University, Brno, Czech Republic; 3 Institute of Clinical Biochemistry and Laboratory Medicine, General University Hospital and First Faculty of Medicine, Charles University in Prague, Prague, Czech Republic; 4 Laboratory of Clinical Immunology, Department of Biomedicine, University Hospital Basel, Switzerland; VU University Medical Center, The Netherlands

## Abstract

Functional deficiency of mannan-binding lectin (MBL) has been associated with adverse pregnancy outcome. Adverse events during pregnancy have also been described in women with autoimmune thyroid diseases (AITD), and thyroid hormones have been shown to influence serum levels of MBL. Therefore, the aim of this study was to analyse the impact of MBL-deficiency on the outcome of pregnancy in relation to the presence of AITD. Almost one year after delivery, we assessed serum MBL levels and *MBL2-*genotypes in 212 women positively screened for AITD in pregnancy. In 103 of these women, we could also measure MBL levels in frozen serum samples from the 9-12^th^ gestational week, obtaining 96 pairs of MBL values (pregnancy vs. follow-up). As controls, 80 sera of pregnant women screened negatively for AITD were used. *MBL2*-genotyping was performed using multiplex PCR. Women with thyroid dysfunction and/or thyroid peroxidase antibodies (TPOAb) had lower MBL levels during pregnancy than controls, (3275 vs. 5000 ng/ml, p<0.05). The lowest levels were found in women with elevated thyroid-stimulating hormone (TSH) levels in the absence of TPOAb (2207 ng/ml; p<0.01 as compared to controls). *MBL2* genotype distribution did not differ between subgroups. At a median follow-up period of 17 months (range: 3–78 months) after delivery, median MBL level had decreased further to 1923 ng/ml (p<0.0001) without significant changes in TSH. In an explorative survey, functional MBL-deficiency was neither linked to a history of spontaneous abortion, nor other obstetric complications, severe infections throughout life/pregnancy or antibiotics use in pregnancy. In conclusion, hypothyroidism during pregnancy is associated with decreased MBL levels, and the levels decreased further after delivery.

## Introduction

Functional deficiency of mannan-binding lectin (MBL) has been associated with adverse pregnancy outcome. Mannan-binding lectin (MBL) is a central component of the lectin pathway of complement, which contributes to the defense against microbial organisms. The high prevalence of point mutations in the *MBL2* gene, the gene encoding for MBL, makes functional MBL deficiency the most common immunodeficiency in humans. In the Czech population, 36.2% of individuals have variant *MBL2* genotypes associated with decreased blood levels of MBL [1]. In childhood, decreased MBL levels may lead to recurrent respiratory tract infections [3]. In adults, however, MBL deficiency seems to be clinically relevant only in immunocompromized or severely ill patients [2]. Independently, functional MBL deficiency has been associated with the occurrence of a number of non-infectious disorders, especially autoimmune diseases such as systemic lupus erythematosus (SLE) or inflammatory bowel disease. Furthermore, functional MBL deficiency seems to be protective in ischemia-reperfusion injury [3].

The role of MBL in human reproduction remains unclear, and results of previous studies are controversial. MBL deficiency of mothers or their offspring has been linked to adverse pregnancy outcomes, such as low gestational age, low birth weight, recurrent miscarriages, increased risk of chorioamnionitis and preeclampsia [4]–[10]. Functional MBL deficiency has also been found to be associated with the production of antiphospholipid antibodies [11], [12] and to predispose to thrombotic events [13], thus increasing the risk of late pregnancy loss [6]. Moreover, MBL takes part in the clearance of circulating apoptotic cell material [14], [15], and it has been proposed that women with low MBL levels may be at risk for excessive inflammatory responses, including the production of autoantibodies due to an altered clearance of apoptotic cell material [4], [6].

Pregnancy seems to have a specific stimulatory effect on the MBL pathway. Previously, several studies have focused on the analysis of serum MBL levels in pregnancy. Kilpatrick et al. found that MBL levels increase only modestly in the first trimester, and that the peak MBL concentrations occur during the third trimester [16]. According to van de Geijn, MBL increases already in the first trimester of pregnancy up to 140% of the baseline level with a sharp decrease after delivery [17]. It was hypothesised that the rise in MBL contributes to normal placentation and ongoing pregnancy, and that it might reflect a shift from adaptive to innate immunity in a state of reduced T-cell function and thus increased susceptibility to infections during pregnancy. They also suggested that higher levels of MBL during pregnancy could be one of the factors responsible for the pregnancy-induced amelioration of certain autoimmune diseases, e.g. rheumatoid arthritis or systemic lupus erythematosus (SLE).

As we have previously shown, serum MBL levels are also linked to thyroid hormones in patients with autoimmune thyroid diseases (AITD) [18] and they correlate with thyroid function in patients with sepsis [19]. AITD are common in pregnancy with about 5.6% of pregnant women suffering from hypothyroidism and 10% being positive for antibodies against thyroperoxidase (TPOAb). Women with AITD encounter adverse pregnancy outcomes similar to those of MBL-deficient women [20]. Therefore, an analysis of MBL levels in pregnancy with regard to the thyroid function seemed to be warranted.

The primary aim for the current study was to assess the relationship between MBL and markers of AITD (TPOAb and increased TSH levels) during and after pregnancy. The secondary explorative aims included evaluation of a possible link between MBL and selected clinical data including occurrence of infections and autoimmunity in pregnancy and the history of obstetric complications.

## Patients and Methods

The study was designed as a retrospective follow-up cohort study and was performed in 2009–2010 in the outpatient section the Third Department of Medicine of the General University Hospital and the First Faculty of Medicine, Charles University in Prague.

All 822 women that screen positive for thyroid disorders during an experimental universal screening program of a total of 5523 consecutive women in the first trimester of pregnancy (9th–12th gestational weeks) performed in Prague and surroundings between 2006 and 2009 were included [21], [22]. Ninety-nine percent of the women were of Caucasian origin. The screening consisted of measurements of thyroid-stimulating hormone (TSH) and TPOAb. In women with pathological values of either TSH or TPOAb, free thyroxine (FT4) was also analyzed. The definition of positivity in screening was based on the reference intervals for TSH and TPOAb in the first trimester pregnancy (see below), i.e. all women with these parameters outside of normal range were regarded as positive for thyroid disorder. Moreover, all pregnant women in the Czech Republic undergo screening for chromosomal aberrations by measurement of Pregnancy-associated plasma protein A (PAPP-A) and Free Beta Human Chorionic Gonadotropin (FbhCG). These parameters were assessed in the same serum sample as thyroid parameters. The laboratory assessment was performed in a single centre (The Institute for Clinical Biochemistry and Laboratory Medicine, General University Hospital in Prague).

At follow-up after delivery we performed a blood test including measurement of TSH, FT4, TPOAb, serum MBL and an analysis of *MBL-2* genotypes. Moreover, the women were asked to fill in a detailed questionnaire. It consisted of 55 questions targeted at the following areas: education, personal and family history of thyroid disease, diabetes, systemic autoimmune disorders (diabetes type 1, psoriasis, vitiligo, pernicious anemia, Crohńs disease, ulcerative colitis, celiac disease, rheumatoid arthritis, SLE, scleroderma, and vasculitis were specified); asthma, atopic eczema, allergies (including specific types of allergy); gynecological history including mode of conception of the current pregnancy; course of the current pregnancy including questions on the occurrence of and hospitalization for infectious diseases and number of prescribed antibiotics during pregnancy; mode of delivery, previous abortions and obstetric complications; and the health of the offspring. Women without access to internet had a possibility to fill a printed form of the questionnaire. Whenever possible (women treated in our department), we also extracted data from the hospital computer database. However, we were not able to validate the adequacy of answers by checking all hospital databases in individual cases, as many women were not followed at our hospital during pregnancy.

Serum samples of 80 pregnant women who tested negative in the screening program (TPOAb negative, normal TSH) were used as controls for serum MBL measurements. These women were not invited for a follow-up and did not fill in the questionnaire.

### Ethics statement

The study was approved by the local ethical committee (General University Hospital, Prague, CZ).All participating women gave written informed consent to the study.

### Biological material

At follow-up after delivery, serum samples were obtained after an overnight fast, and frozen at −20°C until further use. Moreover, we also used frozen serum samples from the time of pregnancy (taken at the same time as serum for screening for thyroid disorders in the first trimester of pregnancy). These samples had been frozen at −70°C until further use. Genomic DNA was isolated from whole blood (EDTA) samples that were drawn at the time of follow-up.

### Serological analysis and reference intervals

MBL was measured using a commercially available Mannan-binding enzyme-linked immunosorbent assay (MBL Oligomer ELISA Kit, BioPorto). This assay uses a mannan coat and development with an anti-MBL antibody, thus measuring MBL as lectin in a functional manner. The lower limit of detection of the test was at 5 ng/ml MBL in serum, the upper cut-off was at 5000 ng/ml. In the statistical analysis, we regarded values >5000 ng/ml as equal to 5000 ng/ml.

Serum MBL levels below 100 ng/ml were classified as “low”, between 100 and 1000 ng/ml as “intermediate”, and over 1000 ng/ml as “normal/high” [23]. All serum MBL measurements were performed in duplicates.

TPOAb were measured using chemiluminiscence on the ADVIA® Centaur™ Analyzer (Siemens). The upper limit of detection of TPOAb was at 10,000 kU/l. Similarly, TSH and FT4 were assayed by ADVIA® Centaur™ Analyzer (Siemens) with chemiluminometry. TSH was determined by sandwich immunoanalysis with direct chemiluminometric technology; for FT4, competitive immunoanalysis with direct chemiluminometric technology was used.

The reference intervals for TSH and TPOAb differed between pregnant and non-pregnant women. As determined by Springer et al., the reference interval for TSH in the first trimester of pregnancy was 0.06 to 3.67 mIU/l and for TPOAb <143 kU/l [22]. The reference intervals for non-pregnant population (women at follow-up) were the following: TSH 0.37-4,0 mIU/l; TPOAb <60 kU/l and FT4: 9.8 to 23.1 pmol/l.

### Genetical analyses

*MBL2* genotypes were determined using multiplex PCR as described previously [1]. The assignment of the haplotypes was based on the strong linkage disequilibrium between the promoter variants and the first exon alleles, and the existence of the frequent haplotypes HYA, LYA, HYD, LYB, LYC, and LXA. We confirmed all LYD haplotypes by a separate long-chain PCR reaction with sequence-specific primers. We considered the following genotypes to be associated with normal/high levels of serum MBL: HYA/HYA, HYA/LYA, HYA/LXA, LYA/LYA and LYA/LXA; while genotypes HYA/HYD, HYA/LYC, HYA/LYB, LYA/HYD, HYA/LYD, LYA/LYB, LYA/LYC, LYA/LYD, and LXA/LXA were considered to have intermediate serum MBL levels. Finally, HYD/HYD, HYD/LYB, HYD/LYC, HYD/LYD, LYB/LYB, LYB/LYC, LYB/LYD, LYC/LYD, LYD/LYD, LXA/HYD, LXA/LYC, LXA/LYB, and LXA/LYD genotype holders were considered to have low MBL levels.

### Statistics

Statistical analysis was conducted using GraphPad Prism 4 (GraphPad Software, San Diego, CA). All values are expressed as median and range, unless otherwise stated. Nonparametric tests (two-tailed Mann Whitney U test, two-tailed Wilcoxon signed rank test, two-tailed Kruskal-Wallis test and Spearman rank correlation test) were applied throughout with differences being considered significant for p<0.05. For the comparison of the *MBL2* genotypes between the groups, Chi square test and two-tailed Fisher tests were used where appropriate.

## Results

From the 822 women invited after delivery, 239 (29.1%) joined the study. The median interval between delivery and follow-up reached 17 months (range 3–78). Of these 239 women, we obtained serum MBL levels in 212 and MBL2 genotypes in 206 women that were considered as the study population. A questionnaire regarding the family, personal and gynecological history was filled by 192 women. In this group of women, we were able to measure additional MBL serum levels in 103 frozen serum samples from the time of pregnancy, which summed up to 96 pairs of samples (MBL levels in pregnancy vs. follow-up).

Of the 103 women positively screened for thyroid disorders in whom we could measure MBL levels after delivery and during early pregnancy, 86 were positive for TPOAb (12 had TSH elevation, 6 had TSH suppression; 68 were euthyroid). Of the 17 TPOAb-negative ones, 10 had TSH elevation and 7 had TSH suppression. As controls for the MBL measurement in pregnancy, sera of 80 pregnant women who screened negatively for thyroid disorders were used.

Baseline laboratory characteristics of the women included are shown in Table 1 (values obtained during pregnancy).

**Table 1:** Serum levels of MBL and thyroid parameters in pregnant women screened for autoimmune thyroid disorders in the 9-12^th^ gestational wks.

	Positive in screening			Negative in screening
	Total	TPOAb neg.	TPOAb pos.	Total
**MBL (ng/ml)**	3275^**^(1.2 -5000) (n = 103)	2339^**^(1.2-5000) (n = 17)	3425^**^(2.5-5000) (n = 86)	5000 (44 – 5000) (n = 80)
**TSH (mU/l)**	2.68^***^(0.0-88.3) (n = 212)	4.08^***^(0.00-11.53) (n = 54)	2.41^***^(0.0 -88.3) (n = 158)	1.43 (0.18-3.16) (n = 80)
**FT4 (pmol/l)**	13.66^**^(8.0-44.6) (n = 190)	14.11^*^(9.82-44.46) (n = 49)	13.49^*^(8.0-38.64) (n = 141)	14.98 (12.31-19.52) (n = 80)
**TPOAb(kU/l)**	808^***^(9.9-15000) (n = 212)	38.55 (9.9-126) (n = 54)	1415^***^(184-15000) (n = 158)	34 (9-68) (n = 80)

^*^(p<0.05), ^**^(p<0.01), ^***^(p<0.001) (Mann Whitney test). Positivity in screening: TSH<0.06 or >3.67 mIU/l and/or TPOAb>143 kU/l. TSH: thyroid stimulating hormone; FT4: free thyroxine; TPOAb: antibodies against thyroperoxidase. All 212 women included provided a blood sample for MBL analysis after delivery. In 103women, MBL could also be measured in a sample frozen at screening in pregnancy, which summed up to 96 pairs (pregnancy vs. follow-up). Statistical significances of comparison between values in positively vs. negatively screened women are marked by

### MBL levels in pregnancy and at follow-up

**Figure 1:**
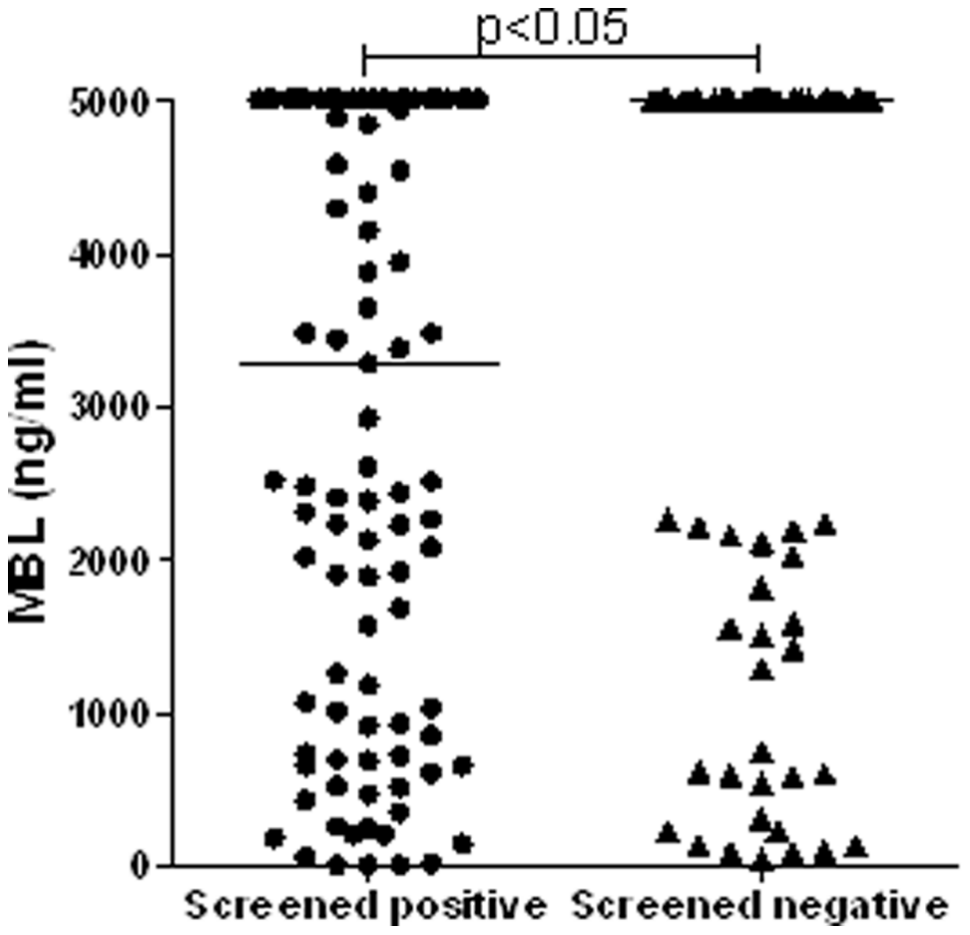
MBL levels in pregnant women with and without autoimmune thyroid disorders. Overall, 103 women tested positively in a screening for AITD performed between the 9^th^ and 12^th^ gestational weeks (grouped together in the left column). They were found to be positive for TPOAb in 86 cases (of these, 12 had TSH elevation, 6 had TSH suppression; the rest was euthyroid). Of the 17 TPOAb-negative ones, 10 had TSH elevation and 7 had TSH suppression. Eighty women were negative for both parameters (right column). Horizontal bars represent median values of serum MBL.

Women, who had abnormal values of the thyroid parameters TPOAb and/or TSH in the first trimester of pregnancy, had significantly lower serum MBL levels (median: 3275 ng/ml), than a control group consisting of women without thyroid disease in pregnancy (5000 ng/ml; p<0.05), as shown in Fig. 1 and Table 1.

**Figure 2:**
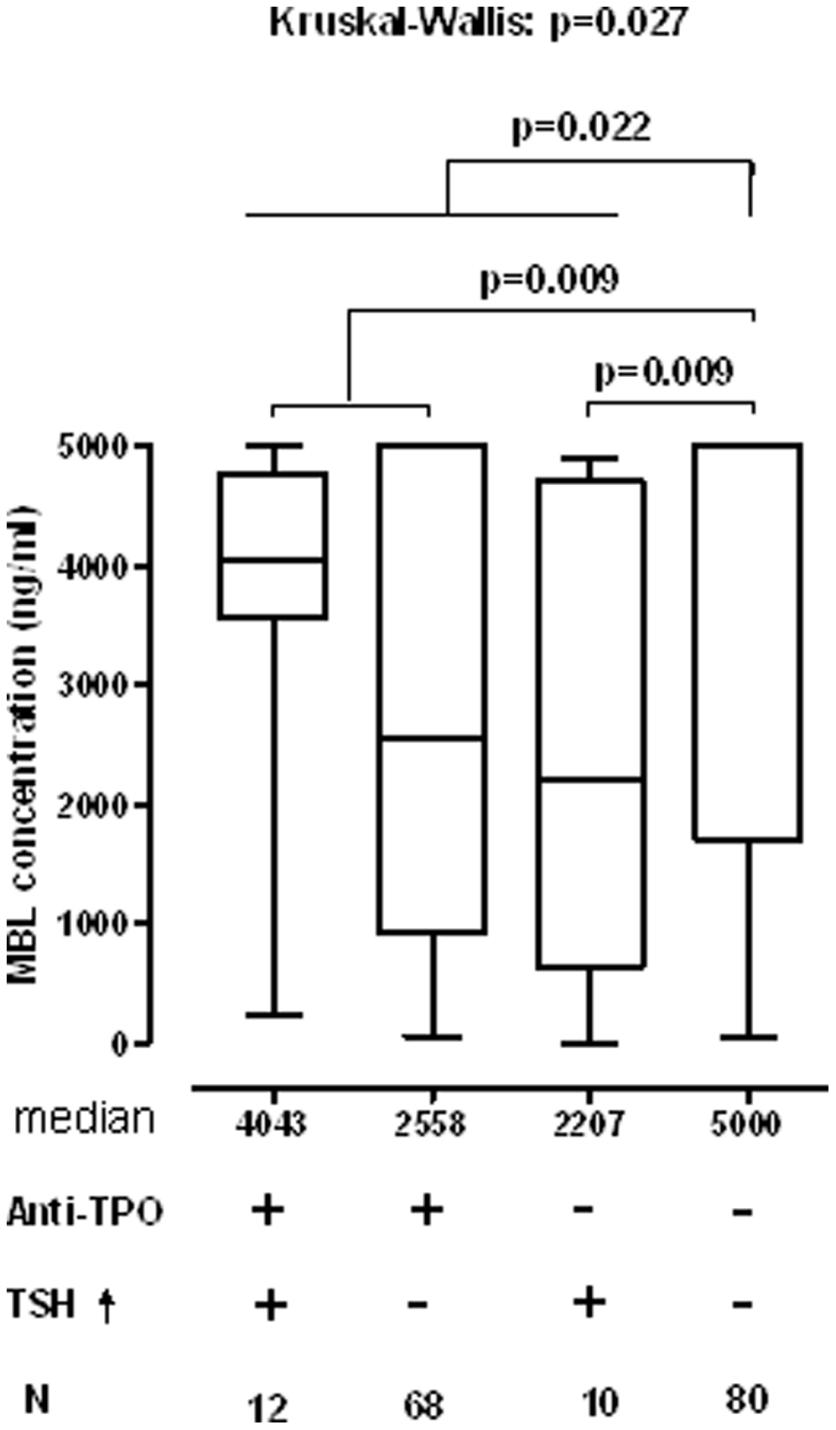
Serum MBL levels in pregnant women according to positivity for TPOAb and the presence of hypothyroidism. The box plots show median, interquartil range and total ranges.

The subgroup analyses showed that there were differences in serum MBL levels in pregnant women screened positively for thyroid disorders according to the presence of hypothyroidism and TPOAb-positivity (Fig. 2). The hypothyroid as well as the TPOAb-positive pregnant women had significantly lower serum MBL levels than the controls. *MBL2* genotype distribution did not differ between subgroups and did not differ from from the general population, as assessed by Skalnikova et al.[1].

Although differences in serum MBL levels between the subgroups of women with thyroid disorders were significant when the absolute values were compared; differences between screening positive versus negative patients that were grouped into low, intermediate and high MBLlevels were not significant but there was a trend for more patients in the intermediate range group (Table 2). Independently, we found a weak positive correlation between serum MBL levels and FbhCG levels in the pregnant control women (r  =  0.232, p  =  0.038).

**Table 2:** Serum MBL levels in pregnant women screened for AITD.

		MBL serum levels	
	Low	Intermediate	High
**Euthyroid TPOAb neg. ( = controls)**	3/80 (3.8%)	12/80 (15%)	65/80 (81.3%)
**Euthyroid TPOAb pos.**	1/68 (0.1%)	17/68 (25%)	50/68 (73.5%)
**All TPOAb pos.**	2/86 (2.3%)	21/86 (24.4%)	63/86 (73.3%)
**Positive in screening total**	5/103 (4.9%)	22/103 (21.4%)	76/103 (73.8%)

–1000 ng/ml and high: >1000 ng/ml. Low serum MBL levels: <100 ng/ml; intermediate: 100

**Figure 3:**
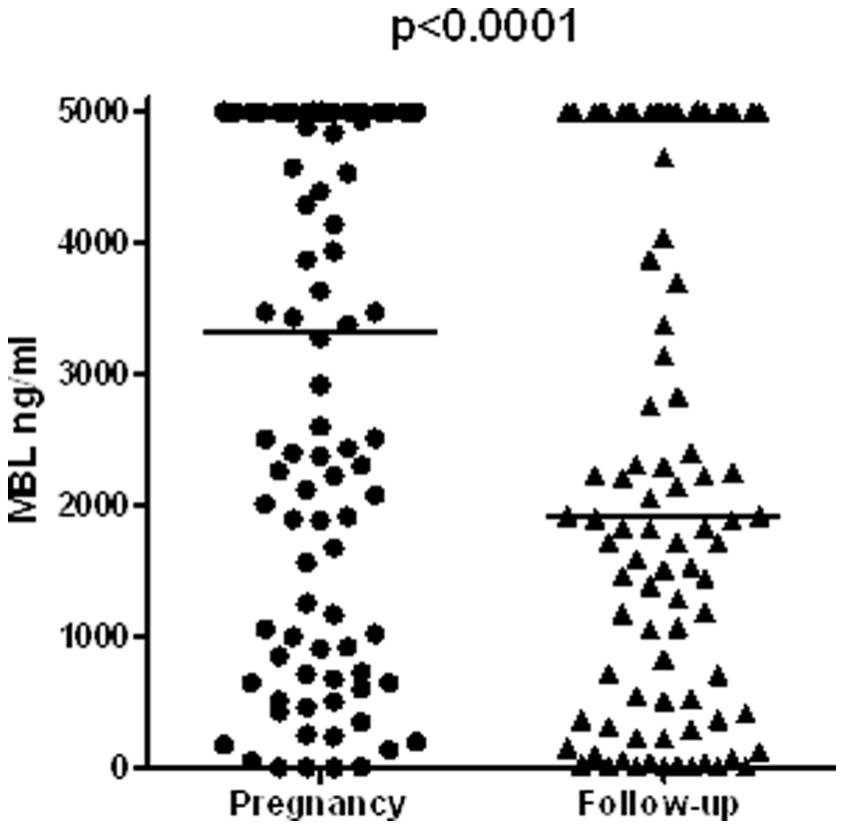
Comparison of serum MBL levels in the first trimester of pregnancy and after delivery in 96 women positive screened for TPOAb and/or thyroid dysfunction in pregnancy. Median interval between delivery and follow-up was 17 months, range 3–78. Wilcoxon signed rank test.

Serum MBL levels markedly decreased after delivery, in comparison to levels measured in the first trimester of pregnancy (Fig. 3). This decrease in MBL was not linked to any significant change in serum levels of TSH or FT4.

### MBL and pregnancy outcome

On the basis of the questionnaires, in an explorative analysis we tried to establish associations between MBL levels and the personal and family history of diseases (Table 3).

**Table 3:** Clinical characteristics of the study group.

Characteristics	Proportion n = 192
**Mean age at pregnancy (years) (median, range)**	31 (21–41)
**University education**	42.2%
**Family history :**	
Thyroid disease	44.0%
Autoimmune diseases (apart from AITD)	11.1%
Asthma	4.9%
**Personal history:**	
AITD (+ TPOAb levels in pregnancy)	74.4%
Autoimmune diseases (apart from AITD)	8.9%
Asthma	4.1%
Atopic eczema	7.4%
Atopic eczema/Asthma	10.7%
Allergy	35.7%
Severe infections requiring hospitalisation	24.2%
**Current pregnancy:**	
Conception with assistant reproduction techniques	12.6%
Conception after > than one and < than two years	46%
Conception after more than two years	10%
Current delivery by caesarean section	35.2%
Premature delivery (<38^th^ gestational week)	17.1%
Min. one hospitalisation in pregnancy (except delivery)	18.2%
Adverse pregnancy outcome (late spont. abortion, medical abortion due to genetic causes, neonatal death at delivery)	1.6%
Mean birthweight (g) (median, range)	3270 (600–4630)
**Infectious complications in the current pregnancy:**	
Min. one treatment with antibiotics	19.2%
Min. one hospitalisation for infectious disease	6.0%
**Previous gynaecological history:**	
Total number of pregnancies (mean) (n)	2 (1–5)
Number of previous spont. abortions (mean) (n)	0.27 (0–3)

AITD: autoimmune thyroid disorders. TPOAb: antibodies against thyroperoxidase. The data were obtained from questionnaires filled by the participating women.

We did not observe any link between MBL levels or *MBL2* genotypes, on the one hand, and the outcome of pregnancy, on the other. Women with subnormal MBL levels (post-delivery MBL levels <1000 ng/ml, and/or *MBL2* genotypes associated with low and intermediate MBL levels) did not have more obstetric complications (premature delivery, spontaneous abortion, caesarean section, preeclampsia) than MBL sufficient women (women with MBL after delivery >1000 ng/ml and/or women with *MBL2* genotypes associated with normal/high MBL levels); similarly, the birth weight of the infants did not significantly differ between the subgroups.

### MBL and history of immune-mediated diseases

In the same explorative analysis MBL-deficient women did not have a higher incidence of severe infections leading to hospitalization before and/or during pregnancy than MBL-sufficient women. Similarly, there were no significant associations between serum MBL levels or *MBL2* genotypes, on the one hand, and prevalence of TPOAb-positivity or personal history of other autoimmune diseases, on the other.

MBL- deficient women (according to both serum MBL levels after delivery and according to the *MBL2* genotypes) had virtually no history of atopic eczema and asthma. On the contrary, women with intermediate levels of MBL had atopy/asthma in more than 20% of cases (Table 4).

**Table 4:** Prevalence of atopy, asthma and allergy with regard to MBL status.

	Assessment of MBL status	Low	MBL Intermediate	High	p – value
**Atopic eczema**	Serum MBL levels	0/18	7/43 (16.2%)	7/107 (6.5%)	0.0596
	MBL2 genotype	0/26	8/47 (17.0%)	5/90 (5.6%)	0.0165
**Atopic eczema and/or**	Serum MBL levels	0/18	9/43 (20.9%)	9/107 (8.4%)	0.0242
	MBL2 genotype	0/26	11/47 (23.4%)	7/90 (7.7%)	0.0320
**Asthma**	Serum MBL levels	0/18	2/43 (4.6%)	5/107 (4.7%)	n/s
	MBL2 genotype	0/26	4/47 (8.5%)	3/90 (3.3%)	n/s
**Allergy**	Serum MBL levels	3/18 (16.6%)	16/43 (37.2%)	40/107 (37.3%)	n/s
	MBL2 genotype	5/26 (19.2%)	18/46 (39.1%)	35/91 (38.4%)	n/s

–1000 ng/ml; high: >1000 ng/ml. MBL2 genotypes represent the allelic variations associated with low, intermediate or high MBL levels. Statistical analysis was performed using the Chi-square test. Serum MBL levels are regarded as low if they are <100 ng/ml; intermediate: 100

## Discussion

We present an analysis of the relationship between serum MBL levels/*MBL2* genotypes and a number of clinical characteristics in a well-described cohort of pregnant women screened positive for thyroid disorders in the first trimester of pregnancy.

In accordance with van de Gein [17], we have found that MBL is strongly increased in the first trimester of pregnancy, as compared to follow-up more than one year after delivery. However, we are the first to show that serum MBL levels in pregnancy are influenced by the presence of thyroid disease. Women screened positive for thyroid disorders had significantly lower MBL levels in the first trimester of pregnancy than women screened normal. In contrast to our previous findings in non-pregnant middle-aged population [18], the influence of thyroid disease on MBL levels in pregnancy was present in its most subtle form: even the isolated TPOAb-positivity in euthyroid pregnant women associated with only a minor TSH shift within the reference range led to a significant decrease in serum MBL levels, as compared to TPOAb-negative, euthyroid pregnant women. This effect was independent on the *MBL2* genotype distribution. However, the previously reported correlations between thyroid hormones and MBL levels were lost in pregnancy. It thus appears that serum MBL levels in pregnancy are influenced by a more complex hormonal background. One example could be our finding of a positive correlation between serum MBL and the beta subunit of human chorionic gonadotropin (HCG) in women without thyroid disease. The beta subunit of HCG is specific for HCG and reflects the total HCG levels. The alpha subunit of HCG is nearly identical to the alpha subunit of TSH[24], and HCG is known to have a TSH-like effect in pregnancy leading to transitory gestational hyperthyroidism [25]. Moreover, it is known that MBL production is stimulated by the growth hormone [26], [27]. Thus, we speculate that human placental lactogen, which has a growth-hormone-like activity, could also influence serum MBL levels in pregnancy.

In our explorative study, we cannot confirm the previously reported negative impact of MBL deficiency on the course and outcome of pregnancy. Similarly, we could not unmask a link between MBL deficiency and the history of spontaneous abortions. However, our study was not specifically designed to assess this issue, as women who actually gave birth were invited to participate and we could evaluate their history of spontaneous abortions based on data from questionnaires only. Moreover, our study was underpowered for this evaluation and the results also might have been influenced by the presence of thyroid disease, which per se carries an higher risk for gynecological and obstetric complications [20]. Finally, we did not examine other potential confounding factors, such as prothrombotic mutations, viral infections, and the presence of other autoimmune diseases.

However, when exploring the impact of MBL deficiency on further anamnestic data, we noted that none of the MBL-deficient women (as assessed both by serum MBL <100 ng/ml and *MBL2* genotypes associated with low MBL) had a history of atopic eczema or asthma compared to a 20% prevalence in the group with intermediate MBL levels. This observation is in concordance with studies reporting higher MBL levels in both children and adult patients with asthma and/or allergic rhinitis compared to healthy controls [28]–[31], The increased MBL levels and activity also correlated with peripheral blood eosinophilia in these patients [29], [30]. Interestingly, Staley et al. found even a positive correlation between MBL levels and the severity of asthmatic symptoms in young children [32]. One can speculate that high MBL levels may contribute to increased complement activation via the lectin pathway, and the complement-activated products C3a and C5a may lead to inflammatory sequelae [31]. In addition, MBL deficiency is associated with an increased number of infections in childhood [9], [33]; which in turn might play a protective role against development of atopy/asthma in adulthood. However, the results are conflicting and some studies reported no or even an opposite association of MBL levels with atopy and/or asthma [34]–[37]. Further research is needed to better elucidate the role of MBL in allergic disease development.

In conclusion, our study is the first to show that serum MBL levels in the first trimester of pregnancy are influenced by autoimmune thyroid disease, even in its subclinical form. In addition, we confirmed in a relatively large number of women that serum MBL levels are markedly increased in the first trimester of pregnancy and decline after delivery. This increase in MBL production during pregnancy seems to be influenced by a complex hormonal background, including human chorionic gonadotropin. Finally, we observed in an explorative analysis that MBL-deficient women might have a lower prevalence of atopy/asthma than MBL-sufficient women warranting further studies. In contrast, our data do not support functional MBL-deficiency being associated with obstetric complications, nor with a history of infections before or during pregnancy.
